# Rapid and dramatic responses to dabrafenib and trametinib in BRAF V600E‐mutated lung adenocarcinoma

**DOI:** 10.1002/rcr2.841

**Published:** 2021-08-30

**Authors:** Takayo Ota, Aya Okabayashi, Masahiro Fukuoka

**Affiliations:** ^1^ Department of Medical Oncology Izumi City General Hospital Izumi Japan; ^2^ Department of Dermatology Izumi City General Hospital Izumi Japan

**Keywords:** BRAF mutation, BRAF/MEK inhibitors, lung adenocarcinoma

## Abstract

V‐raf murine sarcoma viral oncogene homologue B1 (BRAF) is a proto‐oncogene that regulates cell proliferation and survival. BRAF V600E‐mutated lung cancer has aggressive characteristics and is resistant to chemotherapies. Combination of BRAF‐specific inhibitor dabrafenib and mitogen‐activated protein kinase kinase (MEK) inhibitor trametinib is the standard treatment for BRAF V600E‐mutated lung cancer. We report a case of BRAF V600E‐mutated lung adenocarcinoma, which presented with respiratory distress due to deterioration of advanced cancer. The tumour responded rapidly and significantly to BRAF/MEK inhibitors, and the patient's symptoms improved within 2 weeks. BRAF/MEK inhibitors are effective treatment in BRAF‐mutated lung cancer even under critical conditions.

## Graphical abstract

Combination of BRAF (V‐raf murine sarcoma viral oncogene homologue B1)‐specific inhibitor dabrafenib and MEK inhibitor trametinib is the standard treatment for BRAF V600E‐mutated lung cancer. We report a case of BRAF V600E‐mutated lung adenocarcinoma, which presented with respiratory distress due to deterioration of advanced cancer. The tumour responded rapidly and significantly to BRAF/MEK inhibitors, and the patient's symptoms improved within 2 weeks.

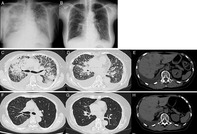

## INTRODUCTION

V‐raf murine sarcoma viral oncogene homologue B1 (BRAF) is a proto‐oncogene that encodes a serine/threonine kinase, which is a component of the mitogen‐activated protein (MAP) kinase pathway that regulates cell proliferation and survival. BRAF mutations are common in a wide variety of cancers. The incidence of BRAF mutations in non‐small cell lung cancer (NSCLC) is 1%–3%, including in Japan.^1^, ^2^ Out of almost 200 functional mutations, the V600E mutation accounts for half of the BRAF mutations found in lung cancer. In this report, we present a case of BRAF V600E‐mutated lung cancer, which showed rapid and dramatic responses to BRAF/mitogen‐activated protein kinase kinase (MEK) inhibitors.

## CASE REPORT

**FIGURE 1:**
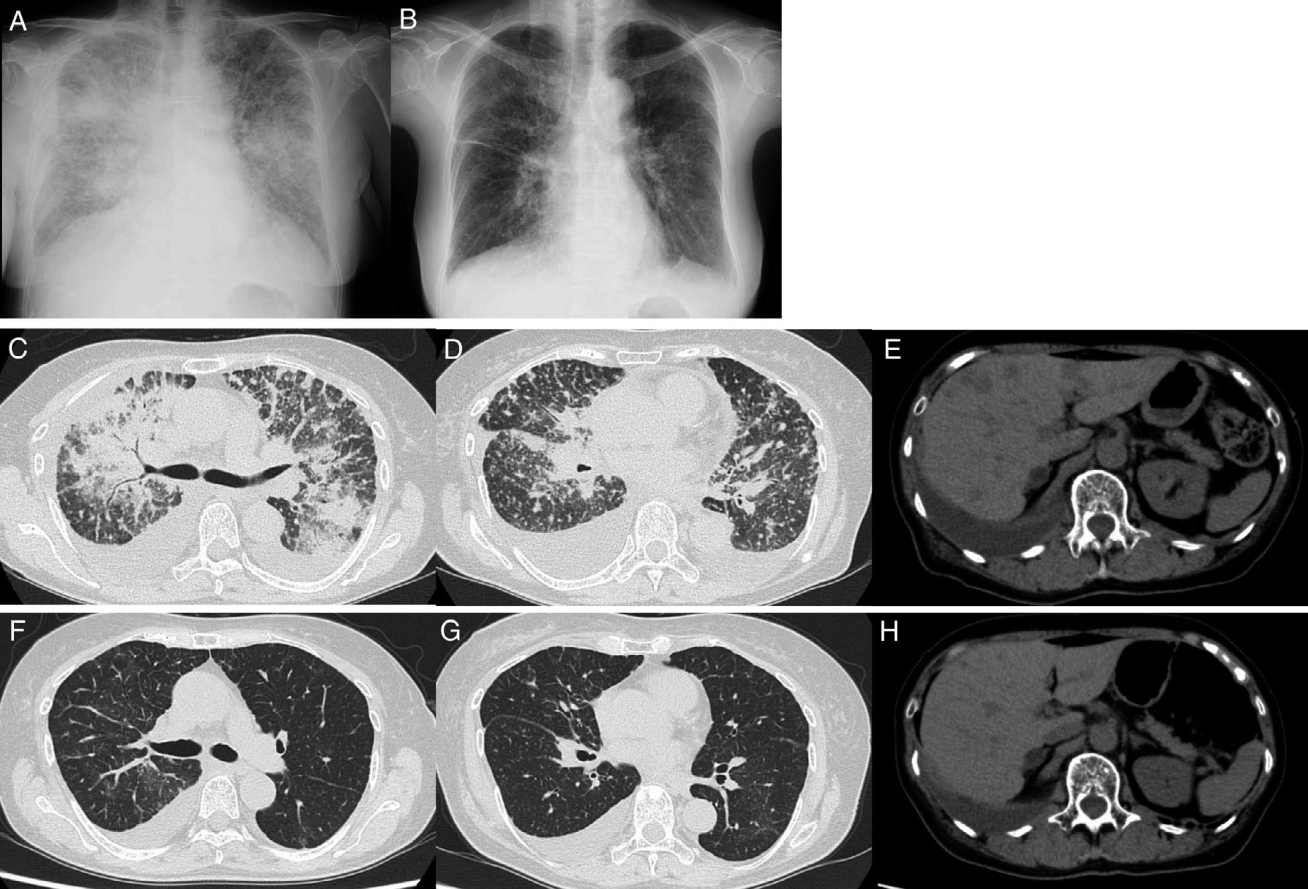
Images showing the patient's response to dabrafenib and trametinib treatment. (A, B) Radiographs of the chest, (A) before the treatment on day 1 and (B) after the treatment on day 15. (C, D, F, G) Computed tomography (CT) of the chest and lung window settings and (E, H) CT of the abdomen and mediastinal window settings. (C–E) Before the treatment on day 1 and (F–H) after the treatment on day 31

In February 2020, a 70‐year‐old woman with no smoking history underwent a chest x‐ray at a yearly medical check‐up, which showed no abnormalities. In September 2020, she noticed a low degree of fever, fatigue and cough. In October 2020, she was diagnosed with advanced lung adenocarcinoma. The patient was referred to the Department of Medical Oncology for treatment. Due to the worsening of her general condition, she was not eligible for conventional chemotherapy. While waiting for the results of molecular testing and programmed death‐ligand 1 (PD‐L1) expression analysis of biopsy tissue, in November 2020, the patient presented at our emergency department with respiratory distress. Her oxygen saturation was 88% with supplemental oxygen at 5 L/min via a cannula. A simple chest x‐ray showed bilateral hazy opacities, and a computed tomography (CT) scan showed multiple lung metastases with severe lymphangitis carcinomatosa and multiple liver metastases (Figure 1). The results of the molecular testing revealed a BRAF mutation resulting in a substitution of valine to glutamate at codon 600 (V600E), but no mutations were found in the epidermal growth factor receptor (EGFR), anaplastic lymphoma kinase (ALK), c‐ROS oncogene 1 (ROS1) or mesenchymal epithelial transition factor receptor (c‐MET) genes. The PD‐L1 expression of the tumour cells was determined to be 1%–49%. Subsequently, on day 1 of therapy, the BRAF V600E‐specific inhibitor dabrafenib (150 mg × 2/day) and the MEK inhibitor trametinib (2 mg/day) were initiated. Soon after the initiation of treatment, the patient's clinical symptoms improved, and on day 11, supplemental oxygen was no longer required. A chest x‐ray on day 13 and a CT scan on day 23 showed significant improvement in the lungs and liver (Figure 1). Regarding the side effects, on day 15, the patient experienced grade 2 maculopapular drug eruption, which was treated with loratadine and betamethasone butyrate propionate ointment. On day 18, the patient was discharged from the hospital. On day 55, a blood test revealed a grade 3 serum amylase increase but no signs of pancreatitis. We reduced the dose of dabrafenib and trametinib. After 6 months, a CT scan showed worsening lung and liver metastases. The patient stopped taking dabrafenib and trametinib. Subsequently, she was treated with one cycle of a combination of chemotherapies and immune checkpoint inhibitors; however, the patient died due to respiratory failure.

## DISCUSSION

In Japan, based on the frequency of driver mutations, their drug availability and drug efficacy, an NGS panel for NSCLC, Oncomine Dx Target Test multi CDx System (SRL, Ltd., Tokyo, Japan), has been approved which examines BRAF V600E mutation, in addition to the most common EGFR mutations, and ALK and ROS1 rearrangements.

The BRAF V600E mutation leads to the constitutive activation of MAP kinase, which increases its oncogenic properties. The BRAF V600E mutation in NSCLC leads to adenocarcinoma with micropapillary features, indicating the aggressive behaviour of the tumours. The BRAF V600E mutation is associated with poor prognosis and lower response rates to platinum‐based chemotherapies in NSCLC.^3^

Targeted therapies against the BRAF V600E mutant protein are designed to inactivate the catalytic activities of BRAF and have been approved for patients with BRAF‐mutated NSCLC. In a phase 2 clinical trial, combination therapy with dabrafenib and trametinib in V600E‐mutated NSCLC showed efficacy.^4^ The response to BRAF/MEK inhibitors polarized into the following two extremes: (i) excellent response and (ii) drug resistance. A dramatic response to BRAF/MEK inhibitors has been reported in several tumours, including glioma and melanoma; however, only a few NSCLC tumours respond favourably to these inhibitors. Dramatic responses to BRAF/MEK inhibitors, in addition to the aggressiveness of the tumours, might be explained by mutations in both the telomerase reverse transcriptase (TERT) promoter and BRAF, whereby the BRAF V600E mutation‐induced MAP kinase pathway predominantly activates the mutant TERT promoter, upregulating TERT expression.^5^ TERT is an enzyme that regulates telomere length, and its overexpression leads to longer telomeres and prevents cellular apoptosis. The TERT promoter mutation rate is approximately 2% in NSCLC.^6^

In summary, we describe a case of BRAF‐mutated lung adenocarcinoma, which recovered quickly with BRAF/MEK inhibitors. The case illustrates it is worth attempting BRAF/MEK inhibitors in BRAF‐mutated lung adenocarcinoma even under critical conditions.

## CONFLICT OF INTEREST

None declared.

## AUTHOR CONTRIBUTION

Takayo Ota drafted the original manuscript. All authors were involved in patient management, and read, critically reviewed and approved the manuscript.

## ETHICS STATEMENT

Appropriate written informed consent was obtained for publication of this case report and accompanying images.
